# A chicken model for studying the emergence of invariant object recognition

**DOI:** 10.3389/fncir.2015.00007

**Published:** 2015-02-26

**Authors:** Samantha M. W. Wood, Justin N. Wood

**Affiliations:** Department of Psychology, University of Southern CaliforniaLos Angeles, CA, USA

**Keywords:** invariant object recognition, *Gallus gallus*, chicks, imprinting, controlled rearing

## Abstract

“Invariant object recognition” refers to the ability to recognize objects across variation in their appearance on the retina. This ability is central to visual perception, yet its developmental origins are poorly understood. Traditionally, nonhuman primates, rats, and pigeons have been the most commonly used animal models for studying invariant object recognition. Although these animals have many advantages as model systems, they are not well suited for studying the emergence of invariant object recognition in the newborn brain. Here, we argue that newly hatched chicks (*Gallus gallus*) are an ideal model system for studying the emergence of invariant object recognition. Using an automated controlled-rearing approach, we show that chicks can build a viewpoint-invariant representation of the first object they see in their life. This invariant representation can be built from highly impoverished visual input (three images of an object separated by 15° azimuth rotations) and cannot be accounted for by low-level retina-like or V1-like neuronal representations. These results indicate that newborn neural circuits begin building invariant object representations at the onset of vision and argue for an increased focus on chicks as an animal model for studying invariant object recognition.

## INTRODUCTION

Humans and other animals can recognize objects despite tremendous variation in how objects appear on the retina (due to changes in viewpoint, size, lighting, and so forth). This ability—known as “invariant object recognition”—has been studied extensively in adult animals, but its developmental origins are poorly understood. We have not yet characterized the initial state of object recognition (i.e., the state of object recognition at the onset of vision), nor do we understand how this initial state changes as a function of specific visual experiences.

Researchers have long recognized that studies of newborns are essential for characterizing the initial state of visual cognition; however, methodological constraints have hindered our ability to study invariant object recognition in newborn humans. First, human infants cannot ethically be raised in controlled environments from birth. Consequently, researchers have been unable to study how specific visual experiences shape the initial state of invariant object recognition. Second, it is typically possible to collect just a small number of test trials from each newborn human. As a result, researchers have been unable to measure newborns’ first visual object representations with high precision.

Here, we describe an automated controlled-rearing approach with a newborn^1^ animal model—the domestic chick (*Gallus gallus*)—that overcomes these two limitations.

### NEWLY HATCHED CHICKS AS A NEWBORN ANIMAL MODEL

Animal models provide a critical tool in the investigation of visual processing machinery. To date, nonhuman primates have been the model of choice for studying invariant object recognition because their visual systems closely mirror our own. Studies of primates have revealed many important characteristics about object recognition, including the nature of its underlying computations and the architecture of its neural substrates (reviewed by DiCarlo et al., 2012; see also Yamins et al., 2014). There is also growing evidence that rats and pigeons may be promising animal models for studying object recognition because they, too, have invariant object recognition abilities (Zoccolan et al., 2009; Soto et al., 2012; Tafazoli et al., 2012; Wasserman and Biederman, 2012; Alemi-Neissi et al., 2013). These animal models enable experimental techniques that are difficult to perform with primates. For instance, rat studies allow the application of a wide range of techniques including molecular and histological approaches, two-photon imaging, and large-scale recordings from multiple brain areas. However, while primates, rodents, and pigeons have many advantages as model systems, these animals are not well suited for studying the *initial state* of object recognition because they cannot be raised in strictly controlled environments from birth^2^.

These three animal models all require parental care. Thus, after birth or hatching, the newborns must be raised in environments that contain a caregiver. Experience with this caregiver could significantly shape the newborn’s object recognition mechanisms by providing clues about which retinal image changes are identity-preserving transformations and which are not. Indeed, studies of monkeys and humans show that object recognition machinery changes rapidly in response to statistical redundancies in the organism’s environment (e.g., Wallis and Bulthoff, 2001; Cox et al., 2005), with significant neuronal rewiring occurring in as little as one hour of experience with an altered visual world (Li and DiCarlo, 2008, 2010). There is also extensive behavioral evidence that primates begin encoding statistical redundancies soon after birth (e.g., Saffran et al., 1996; Kirkham et al., 2002; Bulf et al., 2011). These findings allow for the possibility that even early emerging object recognition abilities (e.g., abilities emerging days, weeks, or months after birth) are learned from experience with objects early in postnatal life.

Analyzing the initial state of invariant object recognition therefore requires a newborn animal model with two characteristics: (1) the animal can develop invariant object recognition abilities and (2) the animal’s visual environment can be strictly controlled immediately after the post-embryonic phase of their life cycle (i.e., to prevent learning from visual object experiences). Chicks meet both of these criteria. First, newly hatched chicks develop invariant object recognition abilities rapidly (Wood, 2013, 2014a). For example, chicks can build a viewpoint-invariant representation of the first object they see in their life (Wood, 2013, 2014a). Chicks also have other advanced object recognition abilities, including the ability to bind color and shape features into integrated color-shape units at the onset of vision (Wood, 2014b). Second, chicks can be raised from birth in environments devoid of objects and caregivers (Vallortigara, 2012; Wood, 2013). Unlike newborn primates, rodents, and pigeons, newly hatched chicks do not require parental care and are immediately able to explore their environment.

In addition, chicks imprint to objects seen soon after hatching (e.g., Bateson, 2000; Horn, 2004). Chicks develop a strong attachment to their imprinted objects, and will attempt to spend most of their time with the objects. This imprinting behavior can be used to test chicks’ object recognition abilities without training (Regolin and Vallortigara, 1995; Bolhuis, 1999; Wood, 2013). Imprinting in chicks is also subject to a critical period (Lorenz, 1937). Once the critical period ends, the chick can be presented with over one hundred test trials without significantly changing the chick’s representation of their imprinted object (e.g., Wood, 2013, 2014a,b). This makes it possible to measure each chick’s first visual object representation with high precision.

Notably, studies of chicks can also inform human visual development because birds and mammals use similar neural mechanisms. At a macro-level, avian and mammalian brains share the same large-scale organizational principles: both are modular, small-world networks with a connective core of hub nodes that includes prefrontal-like and hippocampal structures (Shanahan et al., 2013). Further, avian and mammalian brains have homologous cortical-like cells and circuits for processing sensory information (Jarvis et al., 2005; Wang et al., 2010; Dugas-Ford et al., 2012; Karten, 2013). Although these neural circuits are organized differently in birds and mammals (nuclear vs. layered organization, respectively), they share many similarities in terms of cell morphology, the connectivity pattern of the input and output neurons, gene expression, and function (Saini and Leppelsack, 1981; Karten and Shimizu, 1989; Karten, 1991, 1997; Butler, 1994; Medina and Reiner, 2000; Reiner et al., 2005). For instance, in chicken neural circuitry, sensory inputs are organized in a radial columnar manner, with lamina specific cell morphologies, recurrent axonal loops, and re-entrant pathways, typical of layers 2–5a of mammalian neocortex (reviewed by Karten, 2013). Similarly, long descending telencephalic efferents in chickens contribute to the recurrent axonal connections within the column, akin to layers 5b and 6 of the mammalian neocortex. The avian visual wulst also has circuitry and physiological properties that are similar to the mammalian visual cortex (Karten, 1969, 2013). For example, like the cat and monkey visual cortex, the visual wulst includes precise retinotopic organization, selectivity for orientation, and selectivity for direction of movement (Pettigrew and Konishi, 1976). Together, these studies indicate that birds and mammals use homologous neural circuits to process visual information. Thus, controlled-rearing experiments with chicks can be used to inform the development of vision in humans.

Finally, while chickens have less advanced visual systems than humans, this should not be seen as a problem. When attempting to understand a particular phenomenon, it is often valuable to use the simplest system that demonstrates the properties of interest. Pioneering research in neuroscience and genetics has relied heavily on this strategy—for example, researchers have used *Aplysia* to study the physiological basis of memory storage in neurons (e.g., Kandel, 2007), *C. elegans* to study the mechanisms of molecular and developmental biology (e.g., Brenner, 1974), and *Drosophila* to study the mechanisms of genetics (e.g., Bellen et al., 2010). In a similar vein, the study of newly hatched chicks can offer an important window onto the emergence of high-level visual abilities like invariant object recognition.

### AN AUTOMATED CONTROLLED-REARING APPROACH FOR STUDYING INVARIANT OBJECT RECOGNITION

Historically, newborn subjects’ behavior has been quantified through direct observation by trained researchers. While direct observation has revealed many important insights about human development, this approach has limitations: researchers can only observe a small number of subjects simultaneously, and there are constraints on the resolution of these observations.

Recent technological advances in automated image-based tracking provide a solution to these limitations by allowing researchers to collect large amounts of precise and accurate behavioral data (Dell et al., 2014). Further, image-based tracking uses a digital recording of the animal’s behavior, which maintains an objective view of events. This increases the repeatability of analyses, while allowing subjects to be tracked with high spatiotemporal resolution. Finally, and perhaps most importantly, automated approaches eliminate the possibility of experimenter bias (e.g., bias that may occur when coding the subject’s behavior, presenting stimuli to the subject, or deciding whether to include the subject in the final analysis).

To study the initial state of invariant object recognition, we used an automated controlled-rearing approach. This *complete data* controlled-rearing technique allows researchers to raise newly hatched chicks for several weeks within controlled-rearing chambers (for details see Wood, 2013). We use the term *complete data* because the chambers track and record *all* of the chicks’ behavior (9 samples/second, 24 h/day, 7 days/week), providing a complete digital record of each subject’s behavior across their lifespan. This technique produces hundreds of hours of data for each subject, allowing researchers to measure chicks’ emerging visual-cognitive abilities with high precision.

Importantly, our controlled-rearing chambers also make it possible to control all of the chicks’ visual object experiences. The chambers contain no real-world (solid, bounded) objects, and object stimuli are presented to the chick by projecting virtual objects onto two display walls situated on opposite sides of the chamber. Thus, the chicks’ visual object experiences are limited to the virtual objects presented on the display walls.

### THE PRESENT EXPERIMENT

The current study builds on a previous study that examined whether newly hatched chicks can build invariant object representations at the onset of vision (Wood, 2013). In this previous study, chicks were raised for one week in controlled-rearing chambers that contained a single virtual object that could only be seen from a limited 60° viewpoint range. In their second week of life, we then measured whether chicks could recognize the virtual object across a variety of novel viewpoints. The majority of subjects successfully recognized the object across the novel viewpoints, which shows that chicks can build a viewpoint-invariant representation of the first object they see in their life.

**FIGURE 1:**
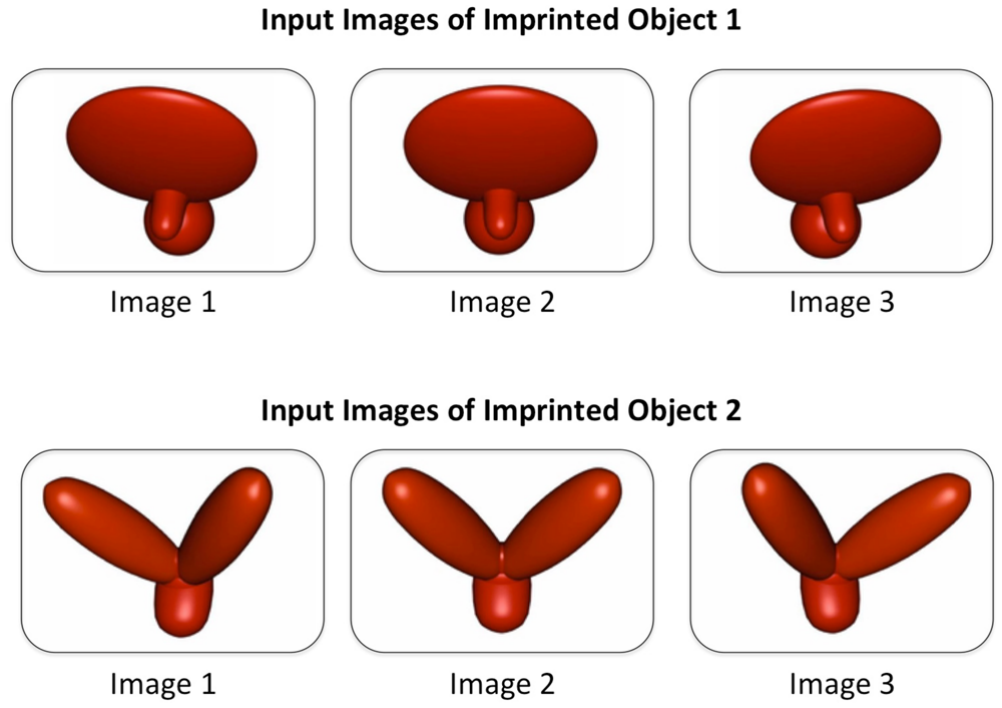
**The three unique images of Object 1 and Object 2 presented to the chicks during the input phase.** Four chicks were presented with Object 1 and six chicks were presented with Object 2. Object 2 served as the unfamiliar object for the chicks that were imprinted to Object 1, and vice versa. The three images changed at a rate of 1 image/second, causing the virtual object to rotate abruptly back and forth through a 30° viewpoint range. Chicks never observed the virtual object (or any other object) from another viewpoint during the input phase.

The present study extends this finding in three ways. First, we significantly reduced the amount of visual object input available to the subjects. In Wood (2013), the chicks were shown a virtual object that moved smoothly over time through a 60° viewpoint range at 24 images/second, whereas in the present study, the chicks were shown a virtual object that moved abruptly over time through a 30° viewpoint range at 1 image/second (see **Figure 1**). Thus, compared with Wood (2013), the chicks in the present study observed a smaller number of unique images of the object (3 unique images vs. 72 unique images), a smaller range of movement (30° viewpoint range vs. 60° viewpoint range), and unnatural (abrupt) vs. natural (smooth) object motion. The abrupt object motion was unnatural because it caused the object’s features to move large distances across the retina instantaneously, breaking the spatiotemporal contiguity of the images. The present study therefore provided a particularly strong test of whether chicks can build invariant object representations from impoverished visual input.

Second, we tested chicks’ object recognition abilities across a systematically varying recognition space. Each chick’s object recognition abilities were tested across 27 different viewpoint ranges; the viewpoint ranges canvassed a uniform recognition space in which the object was rotated -60° to +60° in the azimuth direction and -60° to +60° in the elevation direction (in 15° increments; see **Figure 4**). Thus, we were able to examine whether chicks’ recognition performance varied as a function of the object’s degree of rotation.

Third, we investigated whether chicks’ recognition abilities could be explained by some low-level features of the test animations, by quantifying the similarity between the input images and the test images. We quantified image similarity in terms of both pixel-like similarity and V1-like similarity, akin to previous studies that tested object recognition in adult rats (Zoccolan et al., 2009; Tafazoli et al., 2012).

## EXPERIMENT

### METHODS

#### Subjects

Ten chicks of unknown sex were tested. No subjects were excluded from the analyses. Fertilized eggs were incubated in darkness in an OVA-Easy incubator (Brinsea Products Inc., Titusville, FL, USA). We maintained the temperature and humidity at 99.6°F and 45%, respectively, for the first 19 days of incubation. On day 19 of incubation, the humidity was increased to 60%. The eggs were incubated in darkness to ensure that no visual input would reach the chicks through their shells. After hatching, we moved the chicks from the incubator room to the controlled-rearing chambers in complete darkness. Each chick was raised singly within its own chamber.

#### Controlled-rearing chambers

The controlled-rearing chambers measured 66 cm (length) × 42 cm (width) × 69 cm (height). The floors of the chambers consisted of black wire mesh suspended 1′′ over a black surface by transparent, plexiglass beams. Object stimuli were presented to the subjects by projecting virtual objects onto two display walls (19′′ LCD monitors with 1440 × 900 pixel resolution) situated on opposite sides of the chambers. The other two walls of the chambers were white, high-density plastic. We used matte (non-reflective) materials for both the walls and the floor to avoid incidental illumination. The chambers contained no rigid, bounded objects other than the virtual objects presented on the display walls. See Figure 1 in Wood (2013) for a picture of the chambers.

Food and water were provided *ad libitum* within transparent, rectangular troughs in the ground (66 cm length × 2.5 cm width × 2.7 cm height). Grain was used as food because grain does not behave like a rigid, bounded object (i.e., grain does not maintain a solid, bounded shape). All care of the chicks was performed in darkness with the aid of night vision goggles.

The controlled-rearing chambers recorded all of the chicks’ behavior (24 h/day, 7 days/week) with high precision (9 samples/second) via micro-cameras (1.5 cm diameter) embedded in the ceilings of the chambers and automated image-based tracking software (Ethovision XT, Noldus Information Technology, Leesburg, VA, USA). This software calculated the amount of time each chick spent within zones (22 cm × 42 cm) next to each display wall. In total, 3,360 h of video footage (14 days × 24 h/day × 10 subjects) were collected and analyzed for the present study.

#### Input phase

**FIGURE 2:**
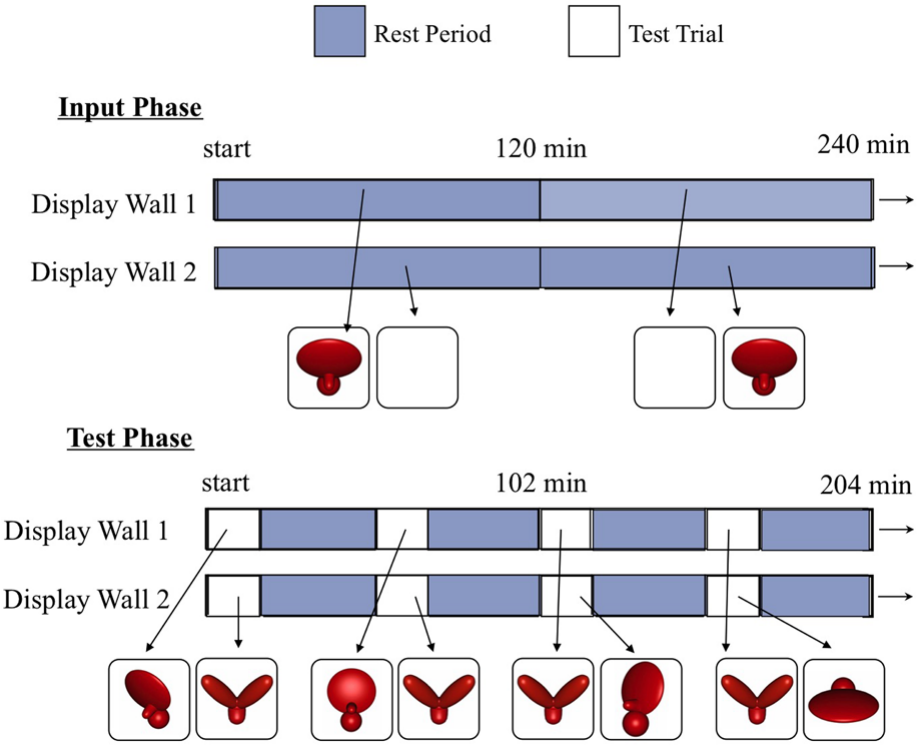
**A schematic showing how the virtual objects were presented on the two display walls during the input phase **(top)** and the test phase **(bottom)**.** During the input phase, chicks observed a single virtual object rotating abruptly back and forth through a 30° viewpoint range. During the test phase, chicks were presented with regularly scheduled test trials. During the test trials, the imprinted object was shown on one display wall and an unfamiliar object was shown on the other display wall. The imprinted object was shown from a variety of novel viewpoints, whereas the unfamiliar object was always shown from the same frontal viewpoint range as the imprinted object during the input phase. This maximized the pixel-level and V1-level similarity between the unfamiliar object and the imprinting stimulus. Thus, to recognize their imprinted object, chicks needed to generalize across large, novel, and complex changes in the object’s appearance on the retina.

During the input phase (the first week of life), chicks were raised in environments that contained a single virtual object. Four chicks were presented with Object 1 and six chicks were presented with Object 2 (see **Figure 1**). The object animations contained just three unique images of the object: a front view and two side views with ±15° azimuth rotations. The images changed at a rate of 1 image/second. From a human adult’s perspective, the objects appeared to undergo apparent motion, rocking back and forth through a 30° viewpoint range along a frontoparallel vertical axis. The virtual object was displayed on a uniform white background, and appeared for an equal amount of time on the left and right display walls. The object switched walls every 2 h, following a 1-minute period of darkness (**Figure 2**).

#### Test phase

During the test phase (the second week of life), we examined whether each chick had built a viewpoint-invariant representation of their imprinted object by using an automated two-alternative forced choice testing procedure. On each test trial, the imprinted object was shown on one display wall and an unfamiliar object was shown on the other display wall. We then measured the amount of time chicks spent in proximity to each object. If chicks successfully recognized their imprinted object, then they should have spent a greater proportion of time in proximity to the imprinted object compared to the unfamiliar object. The imprinted object was shown from 81 different test viewpoints, consisting of all possible combinations of 9 azimuth rotations (-60°, -45°, -30°, -15°, 0°, +15°, +30°, +45°, +60°) and 9 elevation rotations (-60°, -45°, -30°, -15°, 0°, +15°, +30°, +45°, +60°). To equate the direction of object motion across the input and test phases, the 81 viewpoints were organized into 27 different viewpoint ranges, each containing three images. Like the input object animation, each of the 27 test animations showed the imprinted object rotating back and forth ±15° along the azimuth rotation axis. **Figure 4** shows how the 81 individual viewpoints were organized into the 27 test animations.

The unfamiliar object was similar to the imprinted object in terms of its size, color, motion speed, and motion trajectory. Further, on all of the test trials, the unfamiliar object was presented from the same frontal viewpoint range as the imprinted object from the input phase. Presenting the unfamiliar object from this frontal viewpoint range maximized the similarity between the unfamiliar object and the imprinting stimulus. Thus, to recognize their imprinted object, chicks needed to generalize across large, novel, and complex changes in the object’s appearance on the retina. The test trials lasted 17 min and were separated from one another by 32 min rest periods. During the rest periods, we projected the animation from the input phase onto one display wall and a white screen onto the other display wall. The test trials and rest periods were separated by 1 min periods of darkness. On each day of the test phase, chicks were presented with each viewpoint range one time, for a total of 27 test trials per day. Thus, each chick received 189 test trials over the course of the experiment. The 27 viewpoint ranges were presented in a randomized order during each day of the test phase.

### RESULTS

#### Overall performance

To test whether performance was significantly above chance, we used intercept-only mixed effects models (also called “multilevel models”). Since we collected multiple observations from each subject, it was necessary to use an analysis that can account for the nested structure of the data (Aarts et al., 2014). The mixed effects models were performed using R (www.r-project.org). First, we computed the number of test trials in which chicks preferred their imprinted object over the unfamiliar object. The chick was rated to have preferred their imprinted object on a trial if their object preference score was greater than 50%. The object preference score was calculated with the formula:


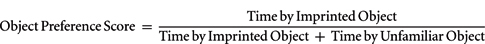


**FIGURE 3:**
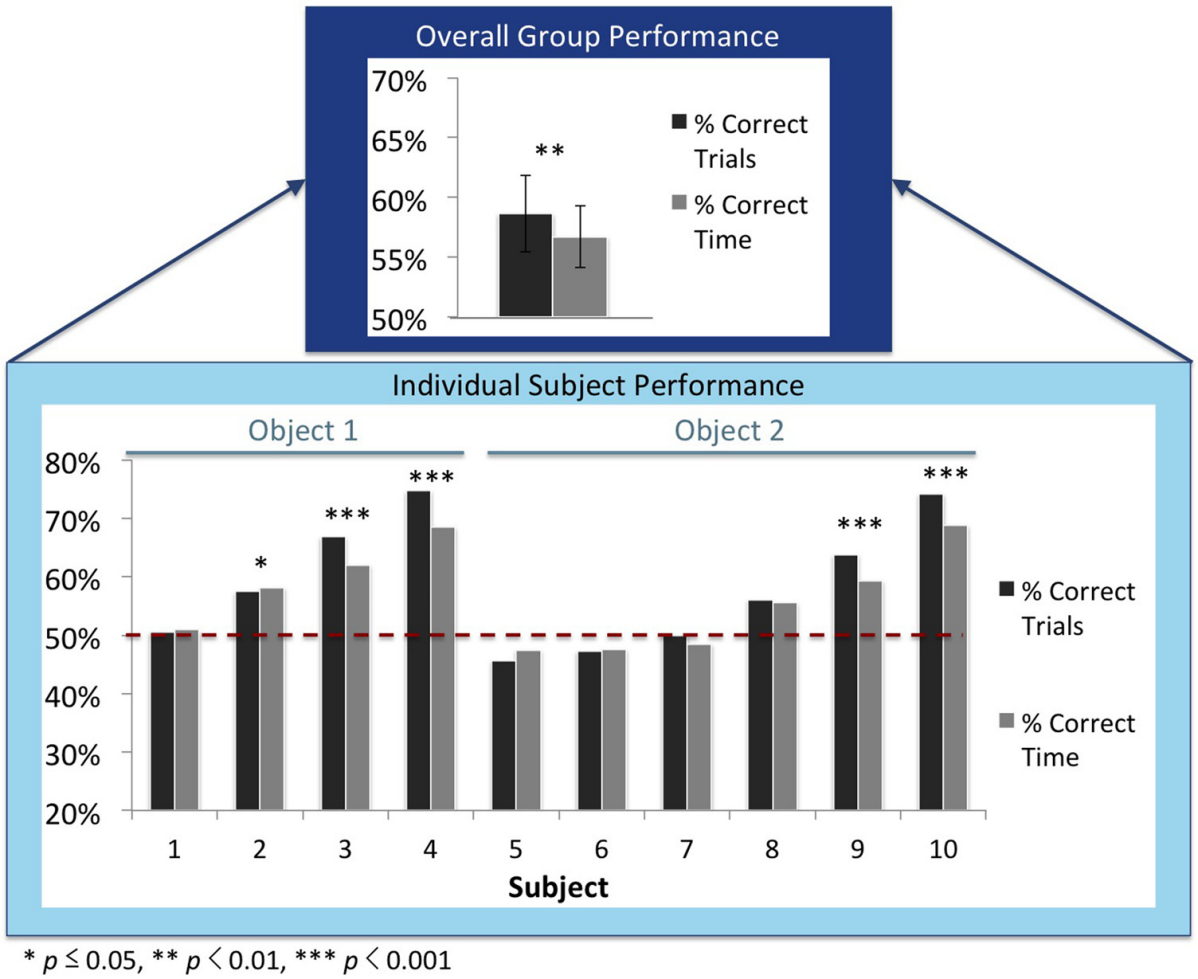
**Recognition performance for the overall group **(top)** and the individual subjects **(bottom)**.** The dark gray bars denote the percentage of correct trials, and the light gray bars denote the proportion of time subjects spent with the imprinted object. These graphs do not include the test trials in which the imprinted object was shown from the familiar viewpoint range from the input phase. The subjects are ordered by performance. The red dashed lines show chance performance (50%). *P*-values denote the statistical difference between the number of correct and incorrect trials as computed through mixed effects models (top graph) and one-tailed binomial tests (bottom graph).

Accordingly, test trials were scored as “correct” when subjects spent a greater proportion of time with their imprinted object, and “incorrect” when they spent a greater proportion of time with the unfamiliar object. Chicks spent more time with their imprinted object on 59% (SEM = 3%) of the test trials (see **Figure 3**).

**FIGURE 4:**
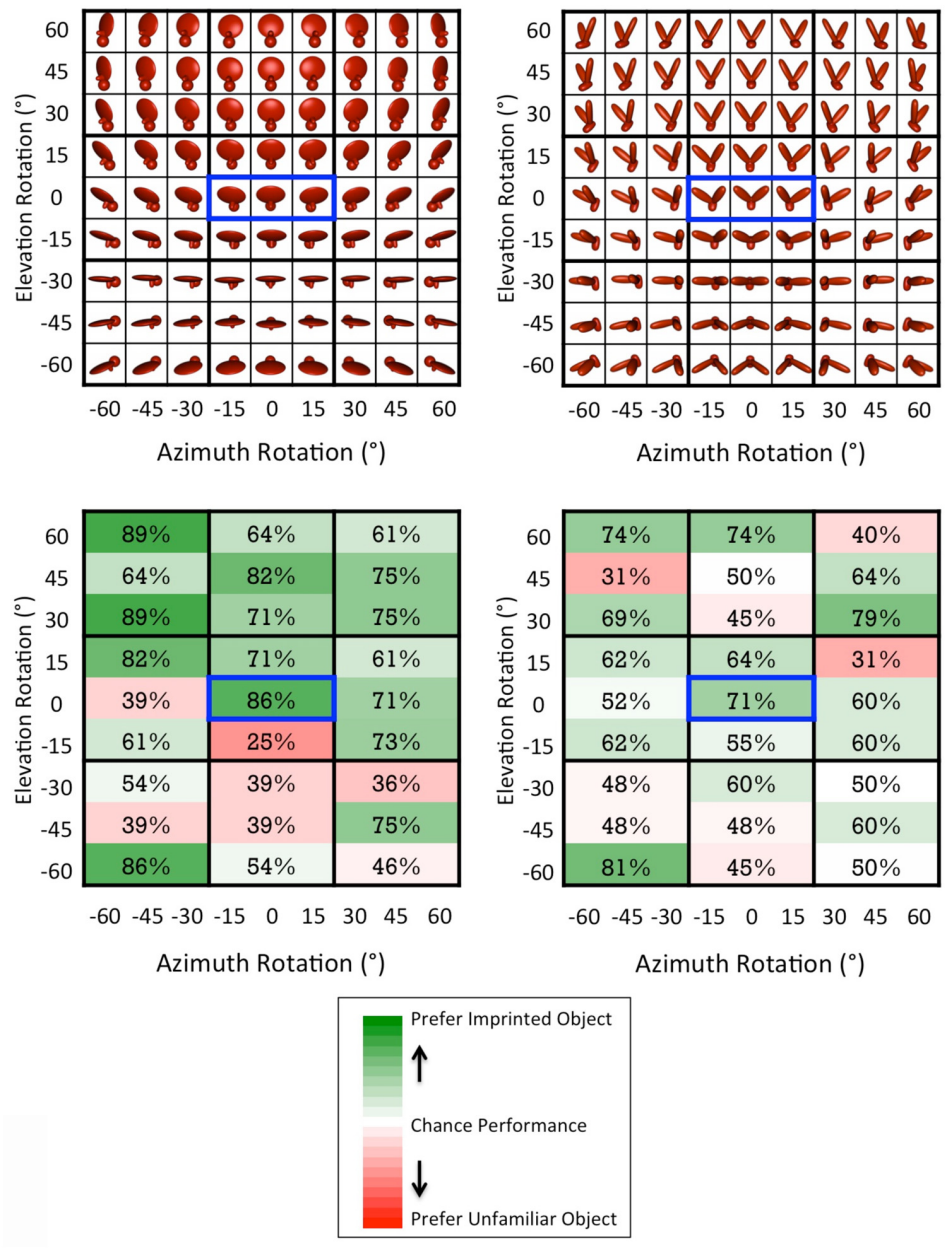
**(Top)** The test viewpoints shown during the test phase. The viewpoint range shown during the input phase is indicated by the blue boxes. **(Bottom)** Chicks’ average percentage of correct trials across the 27 viewpoint ranges. Chance performance was 50%. Each subject received seven test trials for each viewpoint range. Thus, each viewpoint cell in the figure reflects the data from 28 test trials for Object 1 (7 test trials × 4 subjects) and 42 test trials for Object 2 (7 test trials × 6 subjects), for a total of 1,890 test trials across all viewpoint ranges. The color scale reflects the full range of possible performance values (0–100%).

We used a mixed effects logistic regression model (R package lme4) to test whether performance was significantly greater than chance. We fitted the model with test trial outcome (binary: correct or incorrect) as the dependent variable, an intercept as the fixed effect, and a random intercept for the subject-effect. The fixed effect intercept was positive and significant [*b* = 0.394, *z* = 2.857, *p* = 0.004], which indicates that chicks’ recognition performance was significantly greater than 50% (chance performance). Chicks’ recognition performance was also significantly above chance when the analysis did not include the test trials where the imprinted object was shown from the familiar viewpoint range [*b* = 0.365, *z* = 2.636, *p* = 0.008].

Second, we confirmed these results with a similar analysis on the object preference scores (i.e., the proportion of time chicks spent with the imprinted object compared to the unfamiliar object). Because the significance of the intercept indicates whether the intercept is significantly different than 0, we subtracted 50% from each object preference score. Thus, the adjusted object preference scores ranged from -50 to +50%, with an adjusted object preference score of 0 indicating equal time spent with the imprinted object and unfamiliar object. We fitted a linear mixed effects model (R package nlme) with the adjusted object preference score as the dependent variable, an intercept as the fixed effect, and a random intercept for the subject-effect. Again, the fixed effect intercept was positive and significant [*b* = 0.072, *t*(1878) = 3.015, *p* = 0.003], which provides further evidence that chicks’ recognition performance was significantly higher than 50% (chance performance). Chicks’ recognition performance was also significantly above chance when the analysis did not include the test trials where the imprinted object was shown from the familiar viewpoint range [*b* = 0.068, *t*(1808) = 2.828, *p* = 0.005].

With this controlled-rearing method we were able to collect a large number of test trials from each chick. Thus, we were able to examine whether each subject was able to build a viewpoint-invariant representation of their imprinted object. To do so, we computed whether each subject’s performance across the test trials exceeded chance level (using one-tailed binomial tests). Six of the 10 subjects successfully built an invariant object representation [*p*s ≤ 0.05]^3^. When the analysis did not include the familiar viewpoint range from the input phase, 5 of the 10 chicks performed significantly above chance (see **Figure 3**). Thus, many of the chicks successfully built an invariant object representation that generalized across novel viewpoints.

To ensure that all of the chicks successfully imprinted to the virtual object (i.e., developed an attachment to the object), we examined whether the chicks showed a preference for the imprinted object during the rest periods in the test phase. All 10 subjects spent the majority of the rest periods in proximity to the imprinting stimulus [mean = 88% of trials; SEM = 2%; one-tailed binomial tests, all *p* < 10^-9^]. Thus, it is possible to imprint to an object but fail to build a viewpoint-invariant representation of that object (see also Wood, 2013).

#### Correlations of object recognition performance across subjects

**FIGURE 5:**
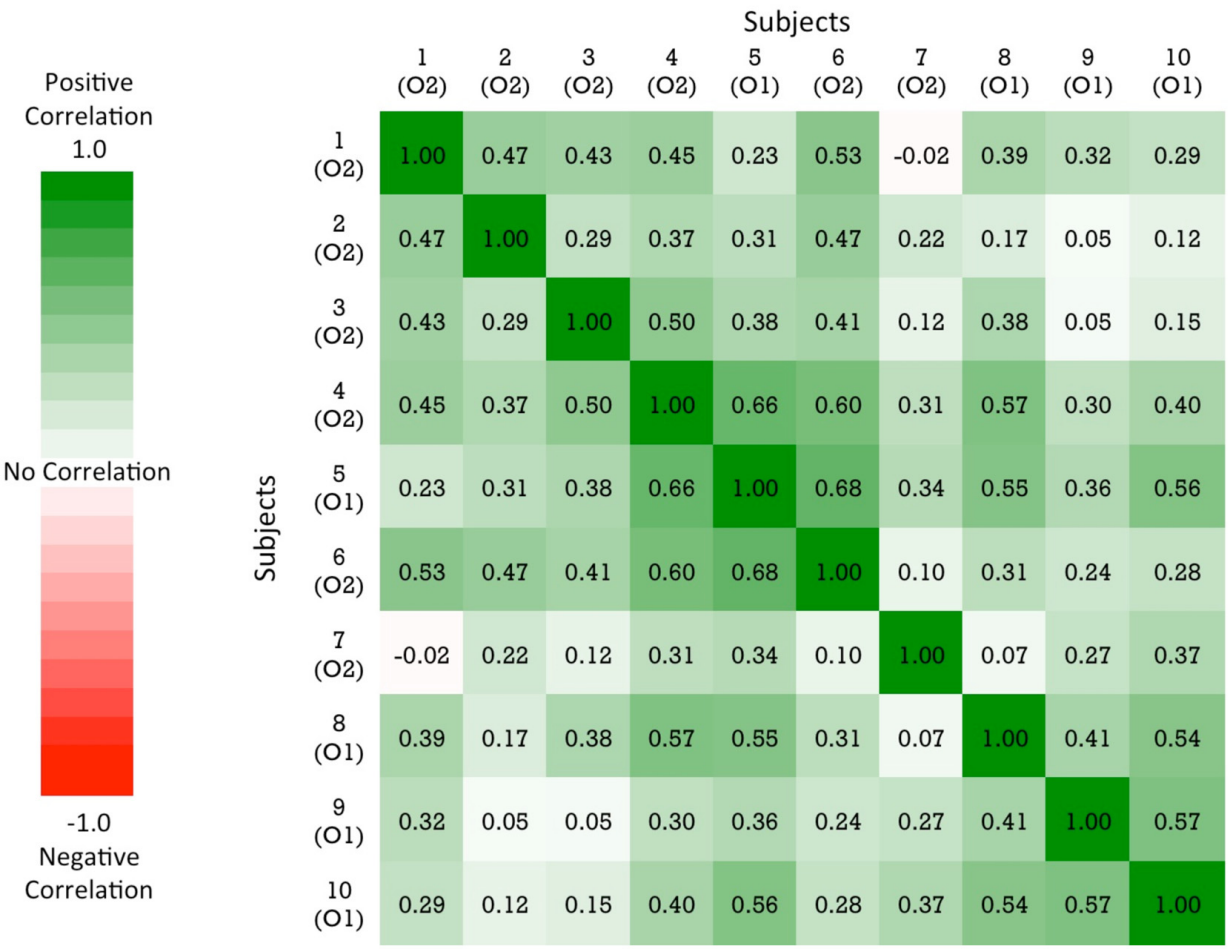
**A similarity matrix showing the correlation in performance for each pair of subjects.** The order of the subjects in the matrix is determined by a hierarchical cluster analysis. The cells are color-coded by correlation value: green values = positive correlation in performance; red values = negative correlation in performance. The color scale reflects the full range of possible correlation values.

As shown in **Figure 3**, there was substantial variation in chicks’ recognition abilities. To examine whether chicks’ recognition abilities were correlated with one another, we measured the correlation in performance across the viewpoint ranges for each pair of chicks. Specifically, we computed the percentage of time spent with the imprinted object for each viewpoint range for each chick. The correlations in performance between all pairs of chicks are shown in **Figure 5**. Performance was highly correlated across the subjects: out of the 45 subject pairs, 44 were positively correlated and only 1 pair was negatively correlated. Overall, the average correlation between subjects was *r* = 0.35 (SEM = 0.03). These correlation values were significantly different from 0 (no correlation), *t*(44) = 8.72, *p* < 0.001. Despite the substantial range of variation in performance across subjects, the chicks’ recognition abilities were nevertheless highly correlated with one another.

#### Analysis of change in performance over time

**FIGURE 6:**
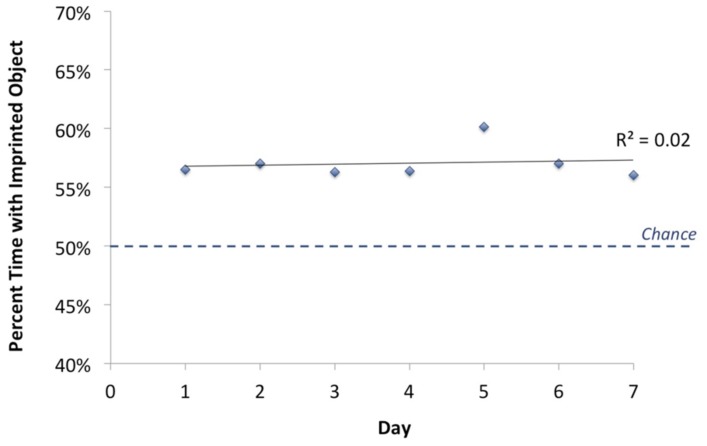
**Change in chicks’ object recognition performance over time.** The graph illustrates group mean performance over the full set of viewpoint ranges shown during the 7 day test phase, computed for the first, second, third, etc., day of testing. Chance performance was 50%. Chicks’ recognition performance did not change significantly across the course of the test phase.

To examine whether recognition performance changed over the course of the test phase, we calculated the percentage of time chicks spent in proximity to the imprinted object versus the unfamiliar object as a function of test day. The results are shown in **Figure 6**. Performance remained stable across the test phase [one-way ANOVA, *F*(6) = 0.224, *p* = 0.968]. Chicks’ recognition behavior was spontaneous and robust, and cannot be explained by learning taking place across the test phase. Chicks immediately achieved their maximal performance and did not significantly improve thereafter.

#### Analysis of viewpoint effects

To test whether recognition performance varied as a function of the degree of viewpoint change, we calculated chicks’ mean object preference scores for each of the elevation viewpoint change magnitudes (i.e., ±60°, ±45°, ±30°, ±15°, 0°). The correlation between the magnitude of viewpoint change and performance did not approach significance [*r* = -0.06, *p* = 0.93]. Thus, when chicks first begin to recognize objects, their performance does not decline with larger changes in viewpoint.

In general, however, chicks’ recognition performance was lower when the object was presented from negative elevation rotations (see **Figure 4**). When the object was presented from negative elevation rotations, a smaller portion of the object was visible to the subject (see **Figure 4**). Thus, chicks’ recognition performance (i.e., the percentage of time spent with the imprinted object versus unfamiliar object) was positively correlated with the number of foreground (object) pixels that were visible on the screen [*r* = 0.41, *p* < 0.01]. One possible explanation for this effect is that the negative elevation rotations occluded discriminative features that were used to recognize the object. For instance, a recent study with adult rats who were trained to distinguish between these same two virtual objects showed that rats built sub-features of objects that were smaller than the entire object (Alemi-Neissi et al., 2013). When these sub-features were occluded with “bubble masks” (Gosselin and Schyns, 2001), rats’ recognition abilities declined. It would be interesting for future studies to use this bubble masking approach with chicks to characterize the specific features used to recognize objects at the onset of vision.

#### Analysis of object stimuli and performance

Did chicks need high-level (invariant) object representations to succeed in this experiment? Previous studies have shown that chicks do not use overall brightness as a low-level cue to distinguish between these two virtual objects (Wood, 2014a), and that chicks’ early emerging invariant object recognition abilities cannot be explained by retina-like (pixel-wise) representations when recognition is tested across more extreme azimuth and elevation rotations (Wood, 2013).

To extend these previous analyses, we quantified the similarity between the input animations and the test animations in two ways. First, we computed the amount of image variation between the input animations and the test animations from a retina-like (pixel-level) perspective. For each animation, we (1) measured the brightness level of each pixel in each of the three unique object images, (2) compared each image from the test animation to each image from the input animation (i.e., by comparing the brightness level of each corresponding pixel across the images and taking the absolute difference), and (3) calculated the average pixel-level difference between the three unique images from the input and test animations (i.e., the first test image was compared to the first, second, and third input image; the second test image was compared to the first, second, and third input image; and the third test image was compared to the first, second, and third input image). Recognition performance (i.e., the object preference scores) did not vary as a function of the pixel-level difference between the input animations and test animations [linear regression: *b* = -7.08 × 10^-8^, *t*(52) = -1.29, *p* = 0.20].

Second, we computed the amount of image variation between the input animations and the test animations from a V1-level perspective. To do so, we used a Gabor measure of similarity with the Gabor jet model: a multi-scale, multi-orientation model of V1 complex-cell filtering developed by Lades et al. (1993). The general parameters and implementation followed those used by Xu and Biederman (2010), which can be downloaded at . For each unique image in each animation, we measured the magnitude of activation values that the image produced in a set of 40 Gabor jets (8 orientations × 5 scales). We measured the dissimilarity between two images by computing one minus the correlation between their Gabor jet activation values. Thus, the dissimilarity between two images could range from 0 (perfect positive correlation) to 2 (perfect negative correlation). Finally, we calculated the average Gabor jet dissimilarity across all three unique images of the animations (i.e., the first test image was compared to the first, second, and third input image; the second test image was compared to the first, second, and third input image; and the third test image was compared to the first, second, and third input image). Recognition performance (i.e., the object preference scores) did not vary as a function of Gabor jet dissimilarity between the input animations and test animations [linear regression: *b* = -0.11, *t*(52) = -1.04, *p* = 0.30].

**FIGURE 7:**
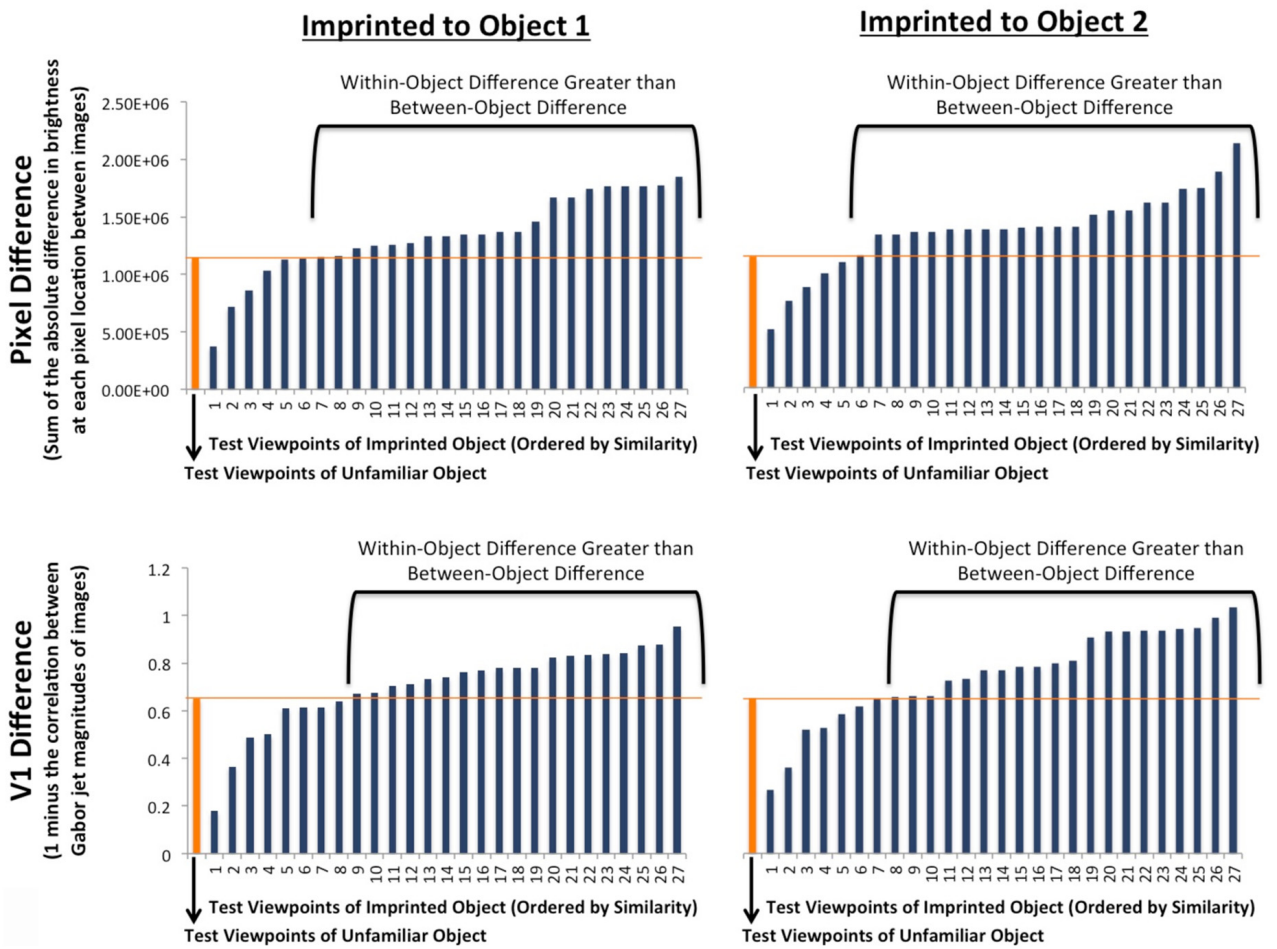
**The average pixel-level and V1-level differences between the three unique images of each test animation and the three unique images of the input animation (i.e., the first test image was compared to the first, second, and third input image; the second test image was compared to the first, second, and third input image; and the third test image was compared to the first, second, and third input image).** The orange bars show the between-object differences (i.e., the difference between the test animation of the unfamiliar object and the input animation of the imprinted object). The blue bars (ordered by similarity) show the within-object differences (i.e., the difference between the test animation of the imprinted object and the input animation of the imprinted object). The top graphs show the differences as measured at the pixel-level, and the bottom graphs show the differences as measured at the V1-level (using Gabor jet magnitudes). Overall, the within-object difference was less than the between-object difference on only 20% (pixel-level) and 28% (V1-level) of the viewpoint ranges (chance performance = 50%). Thus, neither pixel-level nor V1-level representations can be used to reliably predict object identity in this experiment.

Additionally, to confirm that chicks’ recognition performance could not be explained by retina–like or V1–like representations, we tested whether models based on pixel-level or V1-level representations could successfully predict object identity in this experiment. Specifically, we generated a pixel-level model and a V1-level model that predicted object identity based on the image differences between the test animations and the input animation. For each viewpoint range, we measured (1) the difference between the test animation of the imprinted object and the input animation of the imprinted object (within-object difference), and (2) the difference between the test animation of the unfamiliar object and the input animation of the imprinted object (between-object difference; see **Figure 7**). If the within-object difference was smaller than the between-object difference, then the model was “correct” for that viewpoint range. Conversely, if the between-object difference was smaller than the within-object difference, then the model was “incorrect” for that viewpoint range. The retina-like (pixel-level) model was correct on only 20% of the viewpoint ranges, while the V1-level (Gabor jet) model was correct on only 28% of the viewpoint ranges. Unlike the chicks’ recognition performance, which was significantly above chance (50%) levels, both low-level models performed significantly below chance levels [pixel-level intercept-only logistic regression: *b* = -1.36, *z* = -4.04, *p* < 0.0001; V1-level intercept-only logistic regression: *b* = -0.96, *z* = -3.15, *p* = 0.002].

To compare the models’ performance to the chicks’ performance, we computed the average percentage of time chicks spent with the imprinted object versus the unfamiliar object for each viewpoint range. If chicks spent more time, on average, with the imprinted object than the unfamiliar object, then the chicks were “correct” for that viewpoint range. Conversely, if chicks spent more time with the unfamiliar object than the imprinted object, then the chicks were “incorrect” for that viewpoint range. For each model and for the chicks, there were 54 conditions (27 viewpoint ranges × 2 imprinted objects). The chicks were correct on 35 conditions and incorrect on 19 conditions. The pixel-level model was correct on 11 conditions and incorrect on 43 conditions. The V1-level model was correct on 15 conditions and incorrect on 39 conditions. Chi-square tests comparing the number of correct and incorrect conditions for the chicks and the models found significant differences between chicks’ recognition performance and both models’ recognition performance [pixel-level model versus chick performance: *X*^2^(1, *N* = 108) = 21.81, *p* < 10^-5^; V1-level model versus chick performance: *X*^2^(1, *N* = 108) = 14.90, *p* < 10^-3^].

Overall, the within-object difference was greater than the between-object difference, both at the pixel-level and V1-levels. Thus, in principle, chicks could have succeeded in this experiment by preferring the test animation that was the most different from the input animation (i.e., a novelty preference). To test this possibility, we analyzed the test trials in which the imprinted object was presented from the familiar viewpoint range from the input phase. If chicks had a novelty preference, then they should have avoided the imprinted object on the trials in which the test animation of the imprinted object was identical to the input animation of the imprinted object. Contrary to this prediction, chicks spent significantly more time with the imprinted object than the unfamiliar object when the imprinted object was presented from the familiar viewpoint range [logistic mixed effects regression: *b* = 1.514, *z* = 2.794, *p* = 0.005; linear mixed effects regression: *b* = 0.180, *t*(60) = 3.062, *p* = 0.003]. Thus, chicks did not simply have a preference for the novel animation in this experiment.

Together, these analyses indicate that chicks build invariant object representations that cannot be explained by low-level retina-like (pixel-wise) or V1-like neuronal representations. Rather, chicks build selective and tolerant object representations, akin to those found in higher levels of the visual system.

#### Comparison to prior studies

**FIGURE 8:**
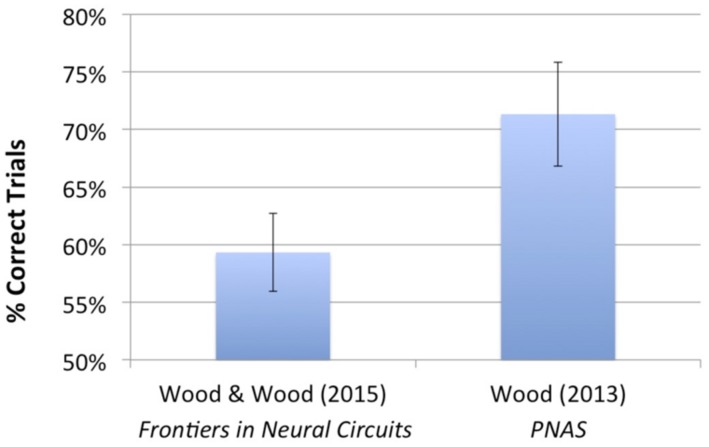
**Average recognition performance for the present study and for Experiment 1 from Wood (2013).** The same two virtual objects were used in both studies. In the present study, the virtual objects moved abruptly over time through a 30° viewpoint range at 1 image/second, whereas in Wood (2013), the virtual objects moved smoothly over time through a 60° viewpoint range at 24 images/second. Thus, compared with Wood (2013), the chicks in the present study observed a smaller number of unique images of the object (three unique images vs. 72 unique images), a smaller range of movement (30° viewpoint range vs. 60° viewpoint range), and unnatural (abrupt) vs. natural (smooth) object motion. Performance was significantly above chance in both studies; however, recognition performance was significantly higher in Wood (2013) than in the present study. Together, these studies show that it is possible to impair chicks’ object recognition abilities by presenting highly impoverished visual object input at the onset of vision.

The virtual objects used in this study were the same as those used in Wood (2013). However, in the current study, each imprinting and test animation only contained three unique images showing the objects rotating abruptly at a rate of 1 image/second, while in Wood (2013), the virtual objects moved smoothly over time through a 60° viewpoint range at 24 images/second. To test whether the impoverished visual stimuli used in the current experiment impaired chicks’ object recognition abilities, we compared performance in the current study to chicks’ performance in Wood (2013). **Figure 8** shows the mean recognition performance from both studies. A one-way ANOVA showed that performance was significantly higher in Wood (2013) than in the current study [*F*(1) = 4.239, *p* = 0.05]. Thus, experience with smooth, continuous object motion over a larger viewpoint range appears to facilitate the development of invariant object recognition. However, additional studies are needed to determine the relative importance of each of these factors (i.e., the number of unique object images, the type of object movement, and the size of the viewpoint range) on chicks’ ability to build invariant object representations.

### GENERAL DISCUSSION

In this study, we examined whether newly hatched chicks can build invariant object representations from highly impoverished visual input (i.e., three images of a single virtual object separated by 15° azimuth rotations). Impressively, many of the chicks successfully built an invariant object representation soon after hatching, which shows that experience with a rich visual world filled with diverse objects is not necessary for developing invariant object recognition. This finding opens up largely unexplored experimental avenues for probing the initial state of invariant object recognition and charting how that initial state changes as a function of specific visual experiences.

#### Implications of our findings and comparison with previous studies

We have previously reported invariant object recognition in newly hatched chicks (Wood, 2013, 2014a); the present study extends this previous research in five ways. First, these results provide an existence proof that newly hatched chicks can build invariant object representations from extremely impoverished visual input. In previous studies (Wood, 2013, 2014a), chicks were shown objects that moved smoothly over time (24 frames/second), thereby presenting large numbers of unique and gradually changing images of the objects. Conversely, in the present study, the object animations were far more sparse (i.e., there were only three unique images of the object), which interrupted the natural temporal stability of the visual object input (i.e., the objects did not change smoothly over time). Thus, the chicks never observed their imprinted object (or any other object) move with smooth, continuous motion. Nevertheless, some of the chicks were able to build an invariant object representation from this impoverished input. For these subjects, three unique images of an object were sufficient input to build an invariant object representation.

Second, these results suggest that it is possible to impair invariant object recognition in newly hatched chicks by presenting abnormally patterned visual input. Although group performance was above chance, performance was significantly lower compared to previous experiments in which the virtual object moved smoothly over time and rotated through a larger viewpoint range (Wood, 2013; see **Figure 8** for comparison of performance between studies). Thus, newborn visual systems appear to operate best over a specific type of patterned visual input. It would be interesting for future studies to characterize the nature of this ‘optimal space’ of visual object input.

Third, these results indicate that invariant object recognition in newly hatched chicks is not subject to the well-documented “viewpoint effect” observed in studies of human adults (i.e., larger viewpoint changes lead to greater costs in object recognition performance; Tarr et al., 1998; Hayward and Williams, 2000). We tested chicks on a wide range of viewpoints, consisting of systematic 15° changes in azimuth and elevation rotations. This allowed us to test whether objects presented from larger viewpoint changes are more difficult to recognize than objects presented from smaller viewpoint changes. We found no significant differences in chicks’ recognition abilities across the larger versus smaller viewpoint changes. Chicks were able to build invariant object representations that generalized beyond the imprinted viewpoint range, but the degree of generalization did not vary as a function of the degree of viewpoint change.

Fourth, we demonstrated that chicks’ object recognition abilities cannot be explained by low-level retina-like or V1-like neuronal representations. Prior experiments have confirmed that chicks’ object recognition abilities could not be explained by overall brightness (Wood, 2014a) or retina-like (pixel-wise) similarity (Wood, 2013, 2014a). Here, we performed additional analyses using simulated Gabor jet activation to measure the V1-like similarity between the input animations and the test animations. We found that chicks’ recognition performance did not vary as a function of the V1-like similarity between the input and test animations. Further, we found that neither a model using pixel-like representations nor a model using V1-like representations was able to successfully predict object identity in this experiment (**Figure 7**). These results indicate that chicks build selective and tolerant object representations, akin to those found in higher-level cortical visual areas (DiCarlo et al., 2012).

Finally, our results provide evidence that invariant object recognition emerges in a consistent manner across different newborn subjects. The chicks’ patterns of recognition performance across the individual viewpoints were strongly correlated with one another (**Figure 5**). This suggests that there are constraints on the development of invariant object recognition in newborn visual systems. However, the data also revealed substantial variation in chicks’ object recognition abilities (see **Figure 3**). Despite being raised in identical visual environments, some chicks were able to recognize their imprinted object robustly across the novel viewpoints, whereas other chicks were not. Future studies could use this controlled-rearing method to further examine both the nature of the constraints on early emerging object recognition abilities and the sources of the individual variation across subjects.

In summary, the present study provides additional evidence that the domestic chick is a promising animal model for studying the emergence of invariant object recognition in a newborn visual system (see also Wood, 2013, 2014a). We have shown how a fully automated controlled-rearing technique can be used to study the initial state of invariant object recognition in newly hatched chicks with high precision. Thus far, our approach indicates that newborn neural circuits are surprisingly powerful, capable of building invariant object representations from impoverished input at the onset of vision.

## SUPPLEMENTARY MATERIAL

The Supplementary Material for this article can be found online at: http://www.frontiersin.org/journal/10.3389/fncir.2015.00007/abstract

## Conflict of Interest Statement

The authors declare that the research was conducted in the absence of any commercial or financial relationships that could be construed as a potential conflict of interest.

## Footnotes

1 The term “newborn” is used to refer to an animal at the beginning of the post-embryonic phase of their life cycle.

2 Rats and mice can be reared in darkness. However, dark rearing prevents complete microcircuit maturation in the visual cortex (Ko et al., 2014), produces abnormalities in local cortical connectivity (Ishikawa et al., 2014), and alters the long-term development of GABAergic transmission (Morales et al., 2002). Further, rats and mice cannot be raised from birth in controlled, lighted environments (i.e., environments devoid of objects and agents). In contrast, chicks can be raised in controlled, lighted environments immediately after hatching. Thus, with chicks, it is possible to examine how patterned visual input drives the emergence of object recognition at the beginning of the post-embryonic phase of the animal’s life cycle.

3 Four of the 10 subjects performed significantly higher than chance level after a Bonferroni correction for 10 independent tests [10 subjects; *p* < 0.005].
