# Supplementary material for: A chicken model for studying the emergence of invariant object recognition
Source: Front Neural Circuits. 2015 Feb 26;9:7. doi: 10.3389/fncir.2015.00007 (PMC4341568; doi:10.3389/fncir.2015.00007)

Supplementary Materials

A chicken model for studying the emergence of invariant object recognition

Samantha M. W. Wood & Justin N. Wood

Table 1. Percentage of correct trials for each subject across the viewpoint ranges

	Subject									
	Imprinted to Object 1				***Imprinted to Object 2***					
**Viewpoint Range**	1	2	3	4	5	6	7	8	9	10
1	85.71%	100.00%	85.71%	85.71%	100.00%	85.71%	57.14%	42.86%	85.71%	71.43%
2	71.43%	100.00%	42.86%	42.86%	71.43%	85.71%	85.71%	57.14%	71.43%	71.43%
3	71.43%	57.14%	85.71%	28.57%	28.57%	28.57%	71.43%	57.14%	28.57%	28.57%
4	57.14%	100.00%	85.71%	14.29%	14.29%	28.57%	28.57%	42.86%	14.29%	57.14%
5	57.14%	100.00%	85.71%	85.71%	71.43%	28.57%	71.43%	42.86%	42.86%	42.86%
6	85.71%	100.00%	57.14%	57.14%	85.71%	71.43%	42.86%	57.14%	71.43%	57.14%
7	100.00%	71.43%	100.00%	85.71%	71.43%	85.71%	85.71%	57.14%	57.14%	57.14%
8	100.00%	85.71%	57.14%	42.86%	57.14%	28.57%	71.43%	28.57%	57.14%	28.57%
9	71.43%	71.43%	85.71%	71.43%	85.71%	85.71%	85.71%	57.14%	85.71%	71.43%
10	85.71%	85.71%	71.43%	85.71%	71.43%	71.43%	100.00%	28.57%	57.14%	42.86%
11	85.71%	100.00%	71.43%	28.57%	71.43%	71.43%	100.00%	14.29%	85.71%	42.86%
12	57.14%	85.71%	57.14%	42.86%	42.86%	0.00%	71.43%	42.86%	14.29%	14.29%
13	42.86%	71.43%	42.86%	0.00%	42.86%	42.86%	71.43%	71.43%	57.14%	28.57%
14	85.71%	57.14%	100.00%	100.00%	100.00%	57.14%	100.00%	57.14%	71.43%	42.86%
15	57.14%	57.14%	100.00%	71.43%	85.71%	71.43%	42.86%	57.14%	42.86%	57.14%
16	71.43%	85.71%	28.57%	57.14%	100.00%	57.14%	100.00%	28.57%	57.14%	28.57%
17	14.29%	42.86%	14.29%	28.57%	85.71%	14.29%	57.14%	42.86%	71.43%	57.14%
18	66.67%	100.00%	66.67%	57.14%	57.14%	57.14%	71.43%	85.71%	42.86%	42.86%
19	71.43%	71.43%	42.86%	28.57%	28.57%	57.14%	85.71%	28.57%	57.14%	28.57%
20	42.86%	42.86%	14.29%	57.14%	71.43%	71.43%	100.00%	28.57%	42.86%	42.86%
21	42.86%	42.86%	28.57%	28.57%	57.14%	57.14%	71.43%	28.57%	57.14%	28.57%
22	57.14%	42.86%	14.29%	42.86%	57.14%	57.14%	57.14%	14.29%	28.57%	71.43%
23	57.14%	57.14%	28.57%	14.29%	42.86%	42.86%	57.14%	42.86%	42.86%	57.14%
24	57.14%	85.71%	71.43%	85.71%	85.71%	85.71%	85.71%	71.43%	0.00%	28.57%
25	85.71%	85.71%	100.00%	71.43%	85.71%	85.71%	100.00%	85.71%	57.14%	71.43%
26	85.71%	57.14%	28.57%	42.86%	42.86%	42.86%	71.43%	42.86%	42.86%	28.57%
27	57.14%	42.86%	28.57%	57.14%	42.86%	42.86%	85.71%	71.43%	28.57%	28.57%

Viewpoint Ranges

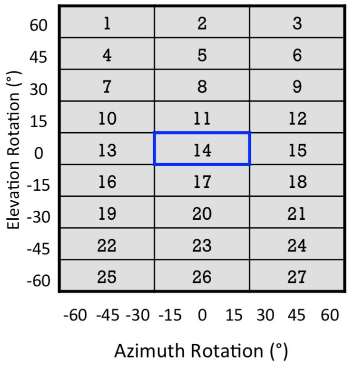

Table 2. Percentage of time spent by the imprinted object compared to the unfamiliar object for each subject across the viewpoint ranges

	Subject									
	Imprinted to Object 1				*Imprinted to Object 2*					
Viewpoint	1	2	3	4	5	6	7	8	9	10
1	79.98%	85.59%	76.19%	83.19%	84.36%	90.51%	66.06%	39.52%	78.34%	67.27%
2	56.66%	91.18%	54.55%	52.50%	59.06%	61.83%	71.52%	60.19%	72.01%	66.60%
3	67.46%	55.20%	79.56%	28.68%	23.48%	26.97%	64.54%	55.00%	32.91%	43.56%
4	50.84%	82.85%	74.17%	22.71%	17.93%	31.44%	49.29%	34.33%	29.11%	38.30%
5	59.42%	88.07%	71.15%	84.19%	63.19%	44.82%	68.62%	50.06%	48.76%	38.34%
6	75.07%	79.08%	72.12%	58.45%	75.53%	63.64%	54.45%	50.66%	56.91%	42.58%
7	90.38%	73.74%	89.63%	84.23%	68.35%	91.20%	67.83%	56.20%	50.34%	51.63%
8	80.91%	77.08%	47.56%	38.19%	57.33%	39.34%	51.99%	32.43%	51.03%	23.32%
9	77.49%	67.92%	75.79%	77.52%	79.27%	74.52%	88.57%	61.52%	73.00%	67.83%
10	73.50%	76.97%	70.97%	72.01%	70.98%	72.31%	94.87%	49.69%	50.12%	52.28%
11	80.71%	80.11%	71.37%	27.57%	68.70%	62.71%	85.86%	21.75%	70.73%	51.70%
12	50.18%	81.57%	51.58%	44.22%	43.93%	9.07%	48.99%	35.71%	28.35%	32.03%
13	27.90%	64.68%	48.83%	0.49%	44.12%	38.69%	69.83%	53.68%	51.43%	37.07%
14	76.39%	49.21%	84.54%	92.44%	84.87%	52.33%	83.80%	35.75%	56.48%	56.45%
15	53.88%	74.64%	90.41%	76.72%	77.74%	66.30%	68.00%	65.01%	44.44%	42.85%
16	67.38%	79.22%	34.86%	57.19%	87.24%	63.16%	92.74%	28.75%	53.50%	49.92%
17	20.16%	39.53%	31.06%	24.98%	76.89%	19.09%	50.47%	45.02%	46.73%	57.84%
18	75.21%	90.64%	66.54%	50.42%	59.15%	60.81%	67.08%	85.16%	44.49%	55.10%
19	57.21%	60.83%	52.21%	41.87%	34.15%	55.30%	67.72%	49.55%	50.85%	33.11%
20	46.63%	48.19%	23.64%	56.72%	61.20%	64.09%	80.58%	48.00%	42.68%	51.01%
21	46.64%	40.50%	39.66%	25.04%	50.56%	57.76%	60.47%	31.81%	41.69%	28.25%
22	54.04%	43.93%	24.32%	42.88%	56.99%	52.14%	52.17%	33.88%	30.50%	76.04%
23	55.07%	47.88%	36.16%	18.97%	43.51%	44.16%	65.39%	39.59%	52.44%	51.39%
24	61.01%	82.10%	78.84%	90.33%	79.97%	90.20%	66.95%	61.86%	30.37%	35.56%
25	75.26%	77.93%	83.36%	74.66%	77.22%	74.75%	83.29%	68.85%	63.59%	70.10%
26	68.53%	47.52%	39.14%	38.36%	46.41%	51.40%	77.45%	32.79%	50.77%	30.52%
27	63.24%	44.66%	30.84%	52.00%	40.80%	50.45%	77.12%	59.07%	21.97%	38.28%

Viewpoint Ranges

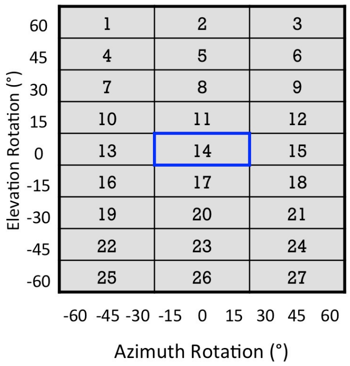

Supplement: Supplementary file 1 [file data_sheet_1.pdf]
